# Mandibular incisors with two canals are associated with the presence of the distolingual root in mandibular first molars: a cone-beam computed tomographic study

**DOI:** 10.1186/s12903-022-02184-4

**Published:** 2022-04-26

**Authors:** Ju-Bin Lee, Min-Seock Seo

**Affiliations:** 1 Department of Conservative Dentistry, Dental College, Wonkwang University, Iksan, Korea; 2 Department of Conservative Dentistry, Wonkwang University Daejeon Dental Hospital, 77 Dunsan-Ro, Seo-Gu, Daejeon, 302-120 Republic of Korea

**Keywords:** Root canal system, Distolingual root, Canal curvature, Mandibular incisors, CBCT

## Abstract

**Background:**

This study aimed to evaluate the root canal configurations and the association between mandibular first molars and distolingual root (DLR) and mandibular incisors using cone-beam computed tomography (CBCT).

**Methods:**

Mandibular first molars and incisors were evaluated using CBCT images of 150 patients. Mandibular first molars were classified according to the presence or absence of DLR. The curvature of the DLR was evaluated using Schneider’s method in buccolingual and mesiodistal orientations. The angle of the mesiolingual–distolingual–distobuccal canal orifices (ML–DL–DB) was evaluated at the pulpal floor level. The mandibular central and lateral incisors were classified as either single canal or two canals. The association between the root canal system of the mandibular first molar and mandibular incisors was evaluated. Data were analyzed using the Chi-square test, Student’s t-test, and odds ratios from binary logistic regression. The significance level was set at 5%.

**Results:**

The frequency of mandibular first molars with DLR was 27.0% and that of mandibular incisors with two canals was 25.8%. The curvature of the DLR was 37.1° in the buccolingual orientation and 10.4° in the mesiodistal orientation. The angle of ML–DL–DB was 79.0°. The presence of two canal systems in mandibular incisors was associated with the presence of DLR in the mandibular first molar (left central incisors: *p* = 0.001, odds ratio = 4.25; left lateral incisors: *p* < 0.001, odds ratio = 3.8; right central incisors: *p* = 0.003, odds ratio = 3.86; right lateral incisors: *p* = 0.001, odds ratio = 3.44) but not with the curvature of the DLR or angle of the ML–DL–DB orifice (*p* > 0.05).

**Conclusions:**

The mandibular first molar showed a high incidence of separate DLRs. It was confirmed that DLR in the mandibular first molar is highly associated with the presence of two-canal system in the mandibular incisors. This association can aid clinicians in successful endodontic treatments.

## Background

Understanding the complexity of the root canal system is essential for successful endodontic treatment. According to a study by Song et al., the main reasons for failure of endodontic treatment are leakage and missing canals [1]. These can result from insufficient knowledge about variations in root canal systems or from leaving untouched areas. Therefore, clinicians should be familiar with the potential aberrant canal anatomy to improve the prognosis of endodontic treatment [2].

The mandibular first molar normally has two roots in the mesial and distal regions [3]. However, in 1844, Carabelli first reported the presence of a separate distolingual root (DLR) [4], later known as Radix Entomolaris [5]. The frequency of DLR varies according to the study population. Studies on Caucasians have reported that the frequency was only 1.7–4.2%, whereas Mongolians had a frequency of 24.5%–27.0 [6–8]. Compared to the distobuccal root, DLR is short and conical [9, 10]. In addition, it has great curvature in the buccolingual direction, which is not detectable on conventional radiographs [10, 11]. As a result, there is a higher possibility of endodontic complications, such as instrument fracture, since cyclic fatigue decreases with an increase in the angle of curvature [12].

Although mandibular incisors usually have relatively simple and uniform root canal morphology, they often have an additional lingual canal that is frequently missed [13, 14]. Unlike the buccal canal, the lingual canal is difficult to detect with periapical radiographs, and it is usually hidden under the cingulum, which requires excessive removal of the dentinal bulge [15]. Failure to manage the lingual canal can lead to endodontic failure [13].

Recently, a lot of research has been conducted on the relationship between DLR in the mandibular first molar and the root canal configuration of neighboring teeth. The second mesiobuccal (MB2) canal in the maxillary first molar [16], complicated root canal configuration of the mandibular first premolar [17], and a c-shaped canal in the mandibular second molar [8] have been investigated. In addition, the correlation between DLR in the mandibular first molar and complicated root canal configuration in the mandibular central and lateral incisors was investigated in Taiwanese patients [18, 19]. However, no studies have been conducted on the relationship between DLR in the mandibular first molar and two canals in both mandibular central and lateral incisors simultaneously in the same subjects. The etiology of this relationship is still unclear, but Papic et al. assumed that it would be related to the timing of root formation [20].

There are diverse methods for evaluating root canal systems, such as radiography, visual examination with extracted teeth, cone-beam computed tomography (CBCT), and micro-computed tomography (micro-CT) [6, 21, 22]. Although the presence of DLR can be detected on conventional radiographs with additional angled views [23], they cannot display the curvature of the root or the canal orifice. Currently, CBCT is widely used as radiographic imaging for endodontic examination and diagnostic applications. Unlike conventional radiography, CBCT can provide detailed information on morphologic analysis using 3-dimentional images and cross-sectional views [24].

The aim of this study was to evaluate the presence of DLR in the mandibular first molar and to analyze the distolingual canal configuration using CBCT. In addition, the association between DLR in the mandibular first molar and two canals in mandibular incisors on the same side was assessed.

## Methods

The protocol for this study was approved by the Institutional Review Board of Wonkwang Dental University Daejeon Hospital (W2104/001-001). The STROBE guidelines were used to confirm the reporting of the study.

To analyze the morphological characteristics of the mandibular first molar, CBCT images were obtained between July 2020 and June 2021 from Sun Dental Hospital (Daejeon, Korea). CBCT images with a large FOV showing mandibular molars and mandibular incisors, which were acquired for the purpose of extraction of the third molar, implant surgery, and orthodontic diagnosis, were selected randomly.

A total of 300 mandibular first molars and 600 mandibular incisors were assessed from 150 patients. The inclusion criteria were as follows: (1) mandibular first molars and mandibular incisors were present bilaterally with complete root formation; (2) absence of root canal filling material; (3) absence of crown, post, and core restorations; and (4) absence of visible external or internal root resorption.

### Evaluation using CBCT

The CBCT images were taken using a CS 9300 (Carestream, Atlanta, GA, USA) at 1-mm slide thickness, 17 × 13-cm field of view, and 300 µm voxel size at 85 kVp and 4 mA. The tomography sections were displayed using ViewRex Viewer (TechHeim, Seoul, Korea). The 3-dimensional (3D) images were reconstructed with CS 3D Imaging software (Carestream). The images were displayed on a 19-inch LCD monitor (Udea, Seoul, Korea) with a 1920 × 1080-pixel resolution in a semi-dark room for assessment. After selecting adequate planes for evaluation, the contrast and brightness were adjusted using the software.

### Morphologic analysis and classification

**Fig. 1:**
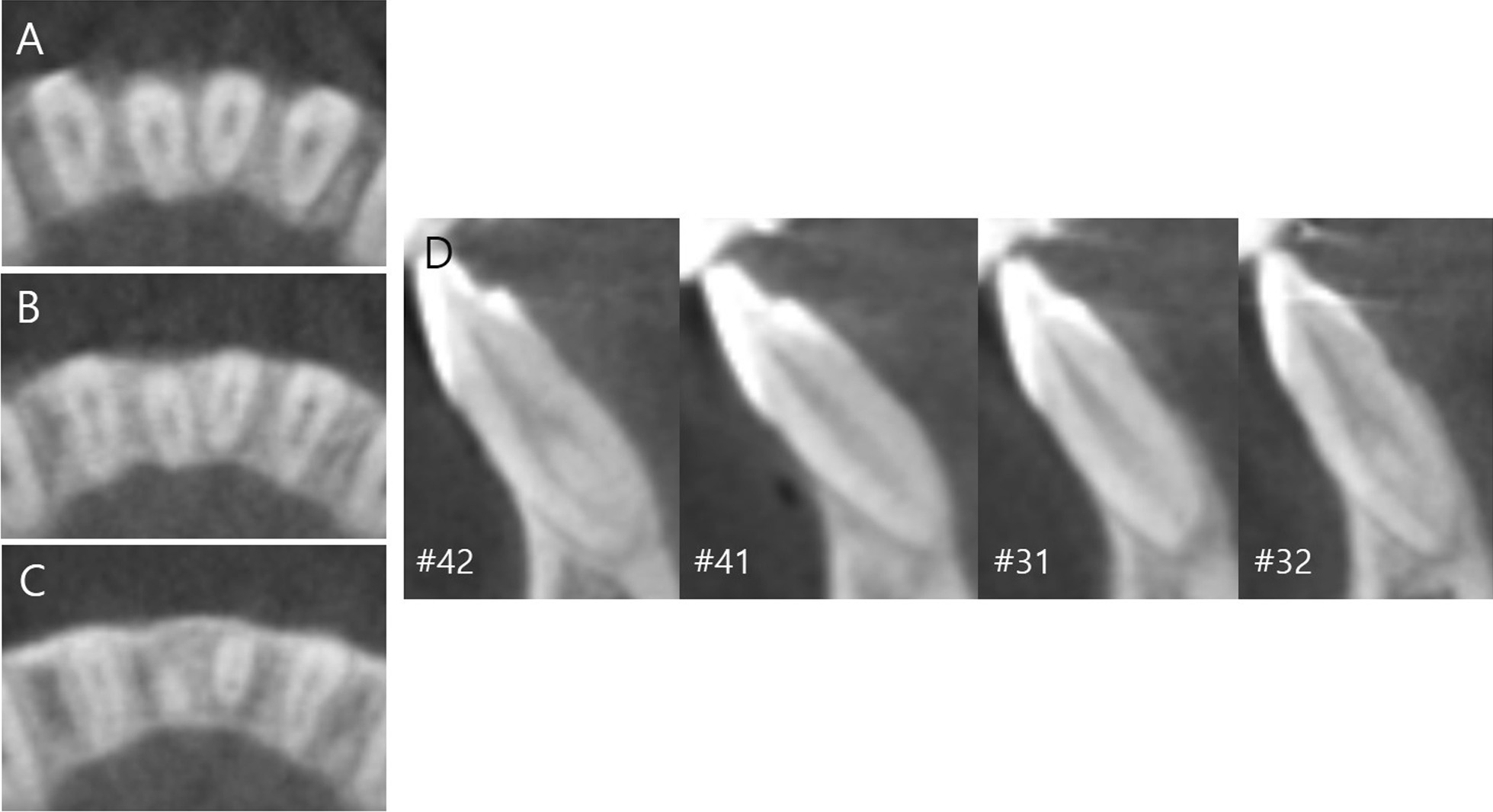
Mandibular central incisors with single canal and lateral incisors with two canal system in a single patient. **A** Axial view (coronal), **B** axial view (middle), **C** axial view (apical), **D** sagittal views

The root canal morphology of mandibular first molars and mandibular incisors was recorded according to patient age, sex, and side. Mandibular first molars were classified according to the presence or absence of DLR. Mandibular incisors were classified as either single canal or two canals. By investigating a series of cross-sectional images from the cementoenamel junction to the apex, mandibular incisors were classified as two canal systems if they had two distinct canals at any single level (Fig. 1).

**Fig. 2:**
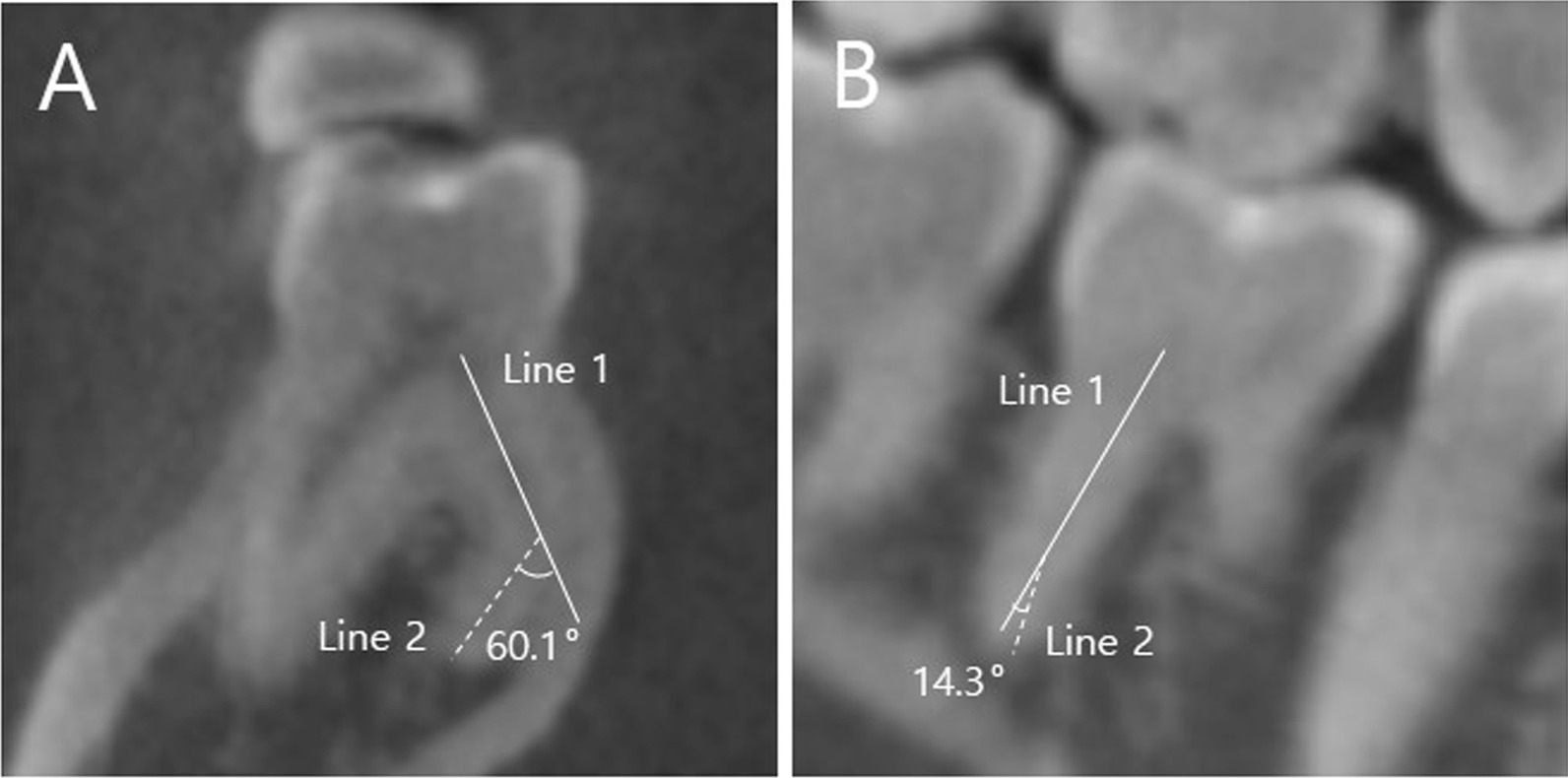
Measurement of disto-lingual (DL) canal curvature. **A** Bucco-lingual orientation, **B** Mesio-distal orientation

**Fig. 3:**
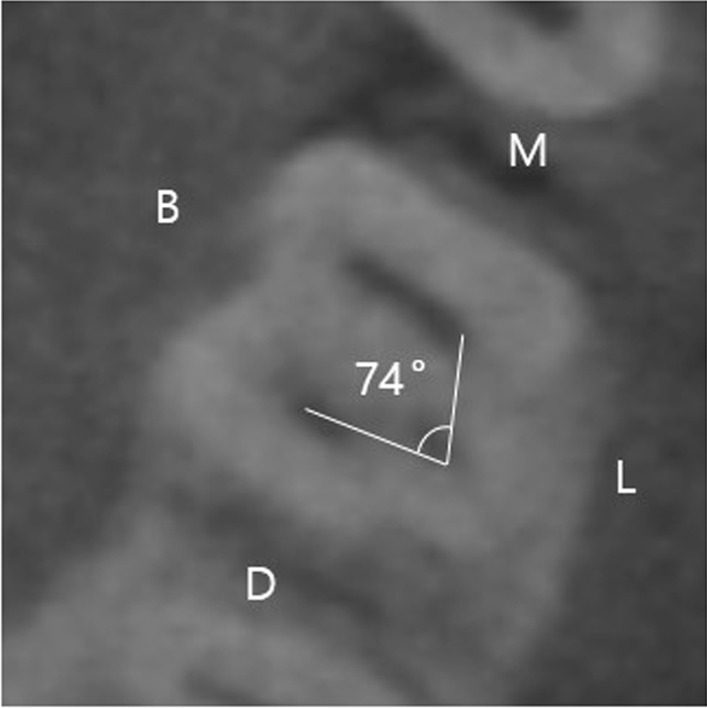
Measurement of angle of mesio-lingual canal orifice–disto-lingual canal orifice–disto-buccal canal (ML–DL–DB) orifice

To investigate the curvature of the DL canal, appropriate cross-sectional views displayed from the canal orifice to the apex in 3D reconstruction programs were selected. Since one specific image cannot display the whole root, the image that showed the root as much as possible was selected. The canal curvature in both buccolingual (B-L) and mesiodistal (M-D) orientations were evaluated using Schneider’s method [25]. Using this method, line 1 connected the long axis of the distolingual (DL) canal from the orifice, and line 2 connected the beginning point of the initial curvature and apical foramen. The angle of curvature was calculated between lines 1 and 2. (Fig. 2). The angle of the mesiolingual–distolingual–and distiobuccal canal orifices (ML–DL–DB) was measured between the center of each orifice at the level of the pulp chamber floor in the axial view (Fig. 3).

### Image validation

Two residents majoring in endodontics (second and third year, respectively) independently evaluated each image twice with a 2-week interval. Inter-examiner calibration was performed before evaluation to confirm the reliability of the data. Kappa values were 0.965 and 0.955 for intra- and interobserver agreement, respectively. In cases of disagreement, a third definitive evaluation was conducted to reach a final consensus by an endodontist with 10 years of experience.

### Statistical analysis

Differences in the frequency of canal configurations according to age, sex, and sides were analyzed using the chi-square test. The association according to the root canal configuration were analyzed using the chi-square test and odds ratios from binary logistic regression. The relationships according to the curvature of the DLR curvature and the angle of the ML–DL–DB orifice were analyzed using the Student’s t-test. All statistical analyses were performed using SPSS Statistics software (Ver 22.0, IBM, Chicago, IL, USA). The significance level was set at *p* < 0.05.

## Results

A total of 300 mandibular first molars and 600 mandibular incisors from 150 patients were evaluated. The patients’ ages ranged from 16 to 37 years, with a mean age of 23.0. The overall frequency of DLR was 27.0% (Table 1). Bilateral and unilateral DLRs were observed in 30 and 21 patients, respectively. The proportion of DLR was 30% on the right side and 24.0% on the left side, but the difference was not significant (*p* = 0.242). Regarding sex differences, the proportion of DLR in males and females was 32.4% and 22.2%, respectively, with a significantly higher preference in males (*p* = 0.046).

**Table 1:** Prevalence (%) of DLR in mandibular first molars

	Left			Right			Total (%)	*p* value
	Male	Female	Total (%)	Male	Female	Total (%)		
Non-DLR	49 (32.7%)	65 (43.3%)	114 (76.0%)	47 (31.3%)	58 (38.7)	105 (70.0%)	219 (73.0%)	p* = 0.242
DLR	22 (14.7%)	14 (9.3%)	36 (24.0%)	24 (16.0%)	21 (14.0%)	45 (30.0%)	81 (27.0%)	
Total	71 (47.3%)	79 (52.7%)	150 (100%)	71 (47.3%)	79 (52.7%)	150 (100%)	300 (100%)	
*p* value	p** = 0.046							

DLR: distolingual root

p*: Chi-square test between the left and right sides. p**: Chi-square test between males and females

The level of statistical significance was set at *p* < .05

In the case of the mandibular first molar with DLR, the mean angles in the B-L and M-D orientations were 37.1° and 10.4°, respectively. The mean angle of the ML–DL–DB orifices was 79.0°, with a standard deviation of ± 6.4° (Table 2).

**Table 2:** The angle of the DLR curvature and the angle of ML–DL–DB orifices in the mandibular first molars

	N	Minimum (°)	Maximum (°)	Mean ± SD (°)
B-L orientation	81	15	70	37.1 ± 12.0
M-D orientation	81	1	42	10.4 ± 6.3
ML–DL–DB orifice	81	65	97.4	79.0 ± 6.4

Among 600 mandibular incisors, the majority had a single canal (74.2%), while two canals were observed in 25.8% (Table 3). The mandibular central incisors and lateral incisors exhibited two canal systems at 15.7% and 36.0%, respectively.

**Table 3:** Root canal configuration of Mandibular Incisors

	Left		Right		Total (%)
	Central incisor	Lateral incisor	Central incisor	Lateral incisor	
Single-canal	126 (21.0%)	95 (15.8%)	127 (21.2%)	97 (16.2%)	445 (74.2%)
Two-canal	24 (4.0%)	55 (9.2%)	23 (3.8%)	53 (8.8%)	155 (25.8%)

The association between DLR in the mandibular first molar and the two canal systems in mandibular incisors was evaluated on the same side (Table 4). The presence of a two-canal system in mandibular incisors was highly dependent on the presence of DLR in mandibular first molars on the same side (*p* < 0.05). The odds ratios were significant for both the central and lateral incisors and both the left and right sides. The left central and lateral incisors were 4.25 and 3.86 times more likely to have two canal systems, respectively, in patients with DLR compared to patients without DLR. The right central and lateral incisors were 3.86 and 3.44 times more likely to have two canal systems, respectively, in the same way. However, there was no significant difference in morphological features, such as curvature of DLR or angle of ML–DL–DB orifice, according to the root canal configuration of both mandibular central and lateral incisors (*p* > 0.05) (Table 5).

**Table 4:** Distribution of DLR of mandibular first molar according to canal system of mandibular incisors on the same side

	Non-DLR	DLR	Total	p* value	Odds ratio (p**)
*Mandibular first molar, left*					
Lateral incisor, left					
Single-canal	81 (54.0%)	14 (9.3%)	95 (63.3%)	< 0.001	3.86 (0.001)
Two-canal	33 (41.8%)	22 (14.7%)	55 (36.7%)		
Central incisor, left					
Single-canal	102 (68.0%)	24 (16.0%)	126 (84.0%)	0.001	4.25 (0.002)
Two-canal	12 (8.0%)	12 (8.0%)	24 (16.0%)		
Total	114 (76.0%)	36 (24.0%)	150 (100%)		
*Mandibular first molar, right*					
Lateral incisor, right					
Single-canal	77 (51.3%)	20 (13.3%)	97 (64.7%)	0.001	3.44 (0.001)
Two-canal	28 (18.7%)	25 (16.7%)	53 (35.3)		
Central incisor, right					
Single-canal	95 (63.3%)	32 (21.3%)	127 (84.7%)	0.003	3.86 (0.004)
Two-canal	10 (6.7%)	13 (8.7%)	23 (15.3%)		
Total	105 (70.0%)	45 (30.0%)	150 (100%)		

p* value obtained using Chi-square test. p** value and odds ratio obtained binary logistic regression The level of statistical significance was set at *p* < .05

**Table 5:** The mean and standard deviation (°) of DLR curvature in B-L, M-D orientation and angle of ML–DL–DB orifice according to root canal configuration of mandibular central and lateral incisors

	Central incisor		*p* value	Lateral incisor		*p* value
	Single canal	Two canal		Single canal	Two canal	
B-L orientation	37.0 ± 12.0	32.8 ± 11.6	0.148	34.6 ± 10.9	36.5 ± 12.8	0.484
M-D orientation	11.1 ± 6.5	8.9 ± 5.5	0.133	11.2 ± 6.7	9.9 ± 5.9	0.373
ML–DL–DB orifice	79.0 ± 6.9	7.9 ± 4.9	0.932	79.0 ± 7.2	79.1 ± 5.8	0.952

*DLR* distolingual root, *B-L* buccolingual, *M-D* mesiodistal, ML–DB–DL mesiolingual–distolingual–distobuccal

Analysis was performed using Student t-test; the level of statistical significance was set at *p* < .05

## Discussion

Mandibular first molars erupt in the oral cavity in the early states and have favorable condition for plaque accumulation [26]. Especially, erupting teeth are susceptible to caries which is related to pre- and post-eruptive maturation of enamel. As a result, mandibular first molars frequently require endodontic treatment and therefore, knowledge of the anatomical morphology is important. While mandibular first molars usually have two roots, many recent studies have reported three roots with separate DLRs [6, 7, 27, 28]. The percentage varies according to the population, with 1.7% in Brazil [27], 2.2% in Portugal [28], and 1.9% in Turkish [6] in the tooth-level analysis. Meanwhile, several studies have suggested that this anatomic feature is associated with racial differences since Mongolians show higher frequency ranging from 24.5 to 27.0% [7, 8, 29, 30]. The present study revealed 27.0%, in accordance with previous studies. An exceptionally high incidence implies that DLR can be regarded as a normal variant among East Asians rather than as an exception [18, 31].

There are still contradictory claims regarding the topologic features of DLR. Although several articles have reported right-side dominance [8, 29], others have found no significant difference [32, 33]. Our study supports this hypothesis. Regarding sex differences, although several studies have suggested no difference [8, 34, 35], others have reported male predilection [29, 36]. We found a significant sex predilection for males.

DLR is relatively short and has severe curvature [9, 21, 27]. In this study, the root curvature was 37.1° in the B-L direction and 10.4° in the M-D direction. The angle of curvature was significantly greater in the B-L direction. This result is similar to that of previous studies. Chen et al. reported that the angle of curvature was 36.4° in the B-L orientation and 9.2° in the M-D orientation using radiographs of extracted teeth [21]. Gu et al. performed similar measurements using micro-CT [9]. They concluded that the angle of curvature was 32.1° in the B-L orientation and 13.9° in the M-D orientation. Since we did not measure the curvature using micro-CT and extracted teeth, the measurement may not be relatively accurate. However, the results were not considerably different. In clinical situations, severe curvature of the DLR may lead to procedural errors, such as file fracture or ledge formation. Therefore, clinicians should take care when negotiating and shaping the root canal.

**Fig. 4:**
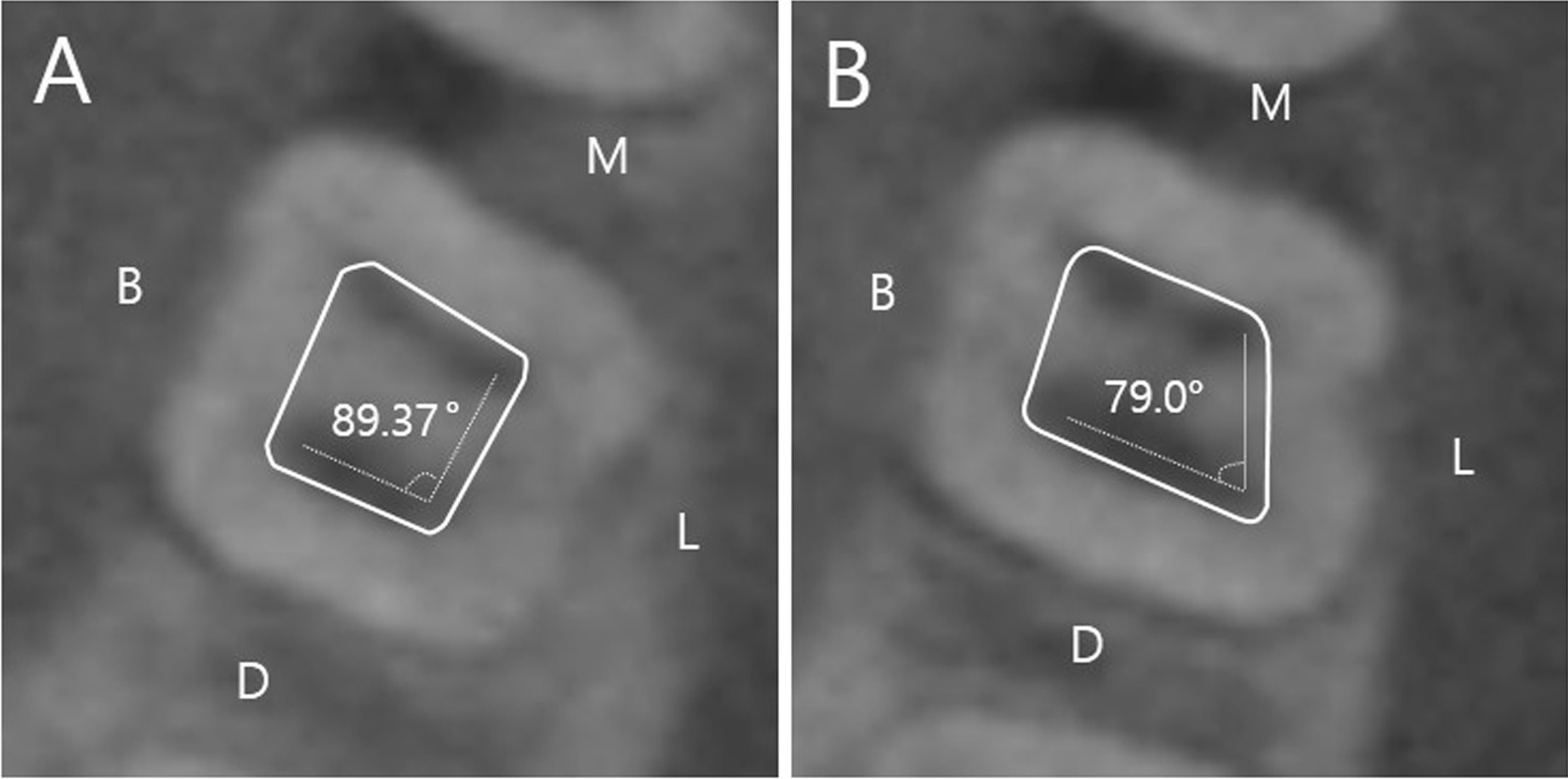
Access cavity form (solid line) according to mesio-lingual canal orifice–disto-lingual canal orifice–disto-buccal canal (ML–DL–DB) orifice (dotted line). **A** Without disto-lingual root. **B** With disto-lingual root

The location of the DL canal orifice influences access cavity design. The orifice of the DL canal is located disto- to mesiolingual from the main canal or canals in the distal end [31]. The present study revealed that the DL canal orifice formed an angle of 79.0° with the DB and ML canal orifices. A previous study reported 75.24°, which is similar to the results of our study [37]. In addition, Jang et al. compared the ML–DL–DB angle in mandibular first molars with DLR (three roots and four canals) and without DLR (two roots and four canals) [38]. They reported that the angle was 77.96° in the mandibular first molar with DLR and 89.37° in the mandibular first molar without DLR. The access cavity is traditionally triangular or rectangular in the mandibular first molars without DLR [31, 37]. However, our results suggest that in case of a mandibular first molar with DLR, the access cavity should be modified to a trapezoidal shape with an acute angle to locate the DL orifice (Fig. 4). Visual ‘map’ in the pulpal floor may help clinicians to locate the entrance.

There are several methods to evaluate the curvature of the root canal, such as periapical radiography, micro-CT, and CBCT [9, 21, 27, 39] Chen et al. were the first to quantitatively measure the curvature of the DLR using extracted teeth and periapical radiographs [21]. They used 21 teeth because of the difficulty in tooth extraction. It is challenging to collect enough extracted teeth because DLR, which has a slender and curved shape, is frequently broken during extraction [10]. Recently, investigations using micro-CT have been reported [9, 27]. Micro-CT is an invaluable tool because it does not require sectioning samples and provides high-resolution images. However, it precludes in vivo use because of its small scan field and the long period of scanning time. In contrast, CBCT facilitates a detailed and accurate assessment of the root canal anatomy without tooth destruction [11]. Additionally, it is possible to perform a cross-sectional investigation of canal morphology with many samples [24]. CBCT is a noninvasive and practical method to routinely assess tooth anomalies in clinical situations.

Mandibular incisors exhibit a high failure rate of endodontic treatment since the second canal is frequently overlooked [13]. In the present study, the overall incidence of the two canals in mandibular incisors was 25.8%. Using CBCT, Liu et al. revealed that two canals were detected in 18.2% of the Chinese population [24]. When considering the differences in the experimental protocol, we used CBCT with a voxel size of 300 μm. However, although Liu et al. used a voxel size of 200 μm with better resolution, they reported a lower incidence than ours. In contrast, one study that used the same 200 μm voxel size exhibited 37.5% of two canals [40]. Although these studies used the same settings for CBCT, the results varied almost two-fold. This may be attributed to the racial and genetic differences. However, the general tendency of lateral incisors to show a higher frequency than central incisors was maintained. This is because lateral incisors usually present long and narrow oval canals, in contrast to central incisors with a rounder canal [33].

Several studies have been conducted to associate DLR in the mandibular first molar and root canal configuration of other teeth [16–19, 41, 42]. Specifically, Wu et al. concluded that DLR in mandibular first molars was significantly associated with the complicated root canal system of mandibular central and lateral incisors, respectively, among Taiwanese [18, 19]. Moreover, our study confirmed association between DLR in the mandibular first molar and two canals in both the central and lateral incisors simultaneously in the same subject. When treating mandibular central incisors and/or lateral incisors for any reason (trauma, TFO, etc.), it is difficult to predict the presence of the lingual canal using preoperative radiographs [18]. However, DLR in the mandibular first molar can be easily detected on conventional radiographs with adequate angulation [23]. As a result, radiographs showing DLR in the mandibular first molar can provide clinicians with a high possibility of lingual canal in any one or both mandibular incisors on the same side. This applies to both the left and right sides. Since the main reason for failure in mandibular incisors is the inability to locate the lingual canal [13], this association can increase the success rate of endodontic treatment while reducing the need to undergo CBCT.

Root canal morphology changes over the course of lifetime with physiological deposition of secondary dentine [43]. According to the study by Martins et al., progressive decrease in Vertucci type I configuration was observed in mandibular lateral incisors. However, mandibular central incisors showed the opposite situation. The mean age of the patients was relatively young with 23.0 years. Although the root canal configuration of the patients may change over the time, Martins et al. stated that the change in the values was not significant in the anterior teeth. According to this conclusion, we could expect that the association might be applied irrespective of the age.

The association of morphological variations between different groups of teeth is highly significant in clinical situations. Nevertheless, the etiology of this relationship remains unknown. Papic et al. revealed the relevance of the MB2 canal of the maxillary first molar and two canals in mandibular lateral incisors [20]. They assumed that this could be related to the timing of root formation. Since mandibular first molar and mandibular incisors have similar developmental stages [44], genetic or other environmental factors may have affected the root canal configuration of both teeth at any stage. Further research on the etiology needs to be conducted.

## Conclusion

According to this study, the mandibular first molar showed a high incidence of separate DLRs in Koreans, which reflects the previous ideas strongly associated with ethnic characteristics. In addition, the association between the presence of two canal systems in the mandibular incisors and mandibular first molars with DLR was confirmed. However, there was no difference in the curvature of the DLR or angle of the ML–DL–DB orifice according to the root canal system of the mandibular incisors. It applies to both the central and lateral incisors on both the left and right sides. Clinicians should bear in mind that there is a high possibility of two canals in mandibular central and lateral incisors if DLR is detected in the mandibular first molar on the same side.

## Data Availability

The datasets used and analyzed during the study are available from the corresponding author upon reasonable request.

## Abbreviations

DLR: Distolingual root
CBCT: Cone-beam computed tomography
B-L: Buccolingual
M-D: Mesiodistal
ML–DL–DB: Mesiolingual–distolingual–distobuccal
